# Screening and purification of natural products from actinomycetes that affect the cell shape of fission yeast

**DOI:** 10.1242/jcs.194571

**Published:** 2017-09-15

**Authors:** Richard A. Lewis, Juanjuan Li, Nicholas E. E. Allenby, Jeffery Errington, Jacqueline Hayles, Paul Nurse

**Affiliations:** 1 Cell Cycle Laboratory, The Francis Crick Institute, 1 Midland Road, London, NW1 1AT, UK; 2 Demuris Ltd, Newcastle Biomedicine Bioincubators, William Leech Building, Newcastle University Medical School, Framlington Place, Newcastle upon Tyne, NE2 4HH, UK

**Keywords:** Fission yeast, Actinomycetes, Natural product, Cell shape, Polyene, Leptomycin B, Cycloheximide

## Abstract

This study was designed to identify bioactive compounds that alter the cellular shape of the fission yeast *Schizosaccharomyces pombe* by affecting functions involved in the cell cycle or cell morphogenesis. We used a multidrug-sensitive fission yeast strain, SAK950 to screen a library of 657 actinomycete bacteria and identified 242 strains that induced eight different major shape phenotypes in *S. pombe*. These include the typical cell cycle-related phenotype of elongated cells, and the cell morphology-related phenotype of rounded cells. As a proof of principle, we purified four of these activities, one of which is a novel compound and three that are previously known compounds, leptomycin B, streptonigrin and cycloheximide. In this study, we have also shown novel effects for two of these compounds, leptomycin B and cycloheximide. The identification of these four compounds and the explanation of the *S. pombe* phenotypes in terms of their known, or predicted bioactivities, confirm the effectiveness of this approach.

## Abstract (precis)

**Summary:** A cell shape-based visual screen of *S. pombe* in the presence of actinomycete-derived bioactivities and an explanation for the phenotypes following identification of the compounds.

## INTRODUCTION

The fission yeast *Schizosaccharomyces pombe* is a rod-shaped unicellular eukaryote that grows by apical extension and divides by medial fission and septation (Mitchison and Nurse, 1985). It has a typical eukaryotic cell cycle and this, together with the highly polarised growth pattern, makes it an excellent organism for studying the mechanisms involved in cell reproduction and the generation of cell form. Both the cell cycle and cell morphology are frequently altered in cancer cells, and mutations that alter these processes play a key role in the development of disease, for example in altering growth control (Beauchamp and Platanias, 2013; Johnson and Halder, 2014), promoting delamination from a epithelial layer (Dekanty et al., 2015) and penetration of surrounding tissues during metastasis (Broustas and Lieberman, 2014). Indeed, morphological features are frequently used in cancer diagnosis. Around 142 genes have been identified in *S. pombe* as being conserved in humans and involved in cancer (Wood et al., 2002; http://www.pombase.org). It follows that compounds capable of perturbing the cell cycle or cell morphology in *S. pombe* may have similar effects in human cells and thus find utility in understanding the cell cycle, cell morphology and cancer, and in the development of anticancer treatments.

Random or site-specific mutagenesis, followed by screening for cells exhibiting aberrant shapes has allowed identification of many genes involved in the cell cycle and cell morphology in fission yeast (Nurse et al., 1976; Nurse and Thuriaux, 1980; Nasmyth and Nurse, 1981; Verde et al., 1995; Snaith and Sawin, 2003). These approaches led to the identification of the conserved Cdc2 (CDK1 in mammals) kinase, its regulatory subunit, the cyclin B Cdc13, and its regulators, the Wee1 tyrosine kinase and the Cdc25 tyrosine phosphatase (Nurse, 1990). Dephosphorylation of Cdc2 at Y15 allows a rise in Cdc2 kinase activity and entry into mitosis; thus, its timing is critical for regulating entry into mitosis (Simanis and Nurse, 1986; Russell and Nurse, 1986, 1987; Gould and Nurse, 1989). Other approaches have utilised near genome-wide deletion collections of essential and/or non-essential genes to identify new genes involved in these processes (Deshpande et al., 2009; Kelly and Nurse, 2011; Navarro and Nurse, 2012; Hayles et al., 2013; Graml et al., 2014).

As an extension of these approaches, we have designed a screen for natural compounds having an effect on the cellular shape of *S. pombe* to identify chemical entities affecting the functions involved in these processes. The development and use of such compounds as research tools to dissect the molecular mechanisms regulating the cell cycle and cell morphology will increase the range of available experimental methodologies. We have previously screened libraries of synthetic compounds produced by combinatorial chemistry (Takemoto et al., 2016; Kawashima et al., 2016), and decided to extend these studies by screening a library of bacterial isolates comprised of actinomycete strains. Actinomycetes are well known for synthesising natural products, for example, bleomycin (Umenzawa et al., 1966) and adriamycin (Arcamone et al., 1969), with anti-eukaryotic cell activities. The rationale behind this approach was that as the mechanisms that determine cell morphology and cell cycle in prokaryotic and eukaryotic organisms are different, and as soil-dwelling bacteria may be competing in the same ecological niche as fungi, these eukaryotic-specific processes could be targets for bacterially synthesised secondary metabolites (Ho and Nodwell, 2016). Thus, evolution would have naturally selected for prokaryotes able to produce antifungal agents, and thus may provide a richer source of ‘hits’ than would be obtained from a library of randomly synthesised molecules.

Phenotypic screens to identify novel bioactive natural products have been used previously; for example, fumagillin was isolated from fungal metabolites, and this compound induced endothelial cell rounding and inhibited angiogenesis (Ingber et al., 1990). Additionally, cell cycle-blocking agents from actinomycetes and fungi have been identified using *S. cerevisiae* as a screening tool (Tsuchiya et al., 2010). A number of screens have also been carried out in *S. pombe* using the viable haploid gene deletion mutant library to examine sensitivity of these mutants to known compounds (Deshpande et al., 2009; Gatti et al., 2011), and a study using wild-type *S. pombe* has looked at the effects of known natural products on cell shape by using FACS (Heisler et al., 2014). The objective of the work described here is to identify bioactive natural products through their effects on *S. pombe* cell shape by visual microscopic screening. We have assayed 657 bacterial strains for their effects on the multidrug-sensitive fission yeast strain SAK950 (Table S1), and report further analysis of the induced phenotype and purification of the active compound from four of these strains as a proof of principle for this experimental approach.

## RESULTS

### Cell shape screening assay

To examine the effects of bioactivities, we used a *S. pombe* strain (SAK950) with increased sensitivity to different compounds due to deletion of seven protein-encoding genes affecting drug efflux (Aoi et al., 2014) (Table S2).

A total of 657 actinomycete bacterial strains (http://demuris.co.uk) were tested for the production of bioactivities affecting the cellular shape of fission yeast (see Materials and Methods for details). We found 420 bacterial strains that led to the formation of a halo around the bacterial plug, indicating the presence of inhibitory bioactive natural products produced by the bacterial strain. For the remaining strains, 237 did not form a halo, and the *S. pombe* strains in the vicinity of the plug showed a wild-type phenotype.

**Fig. 1.**
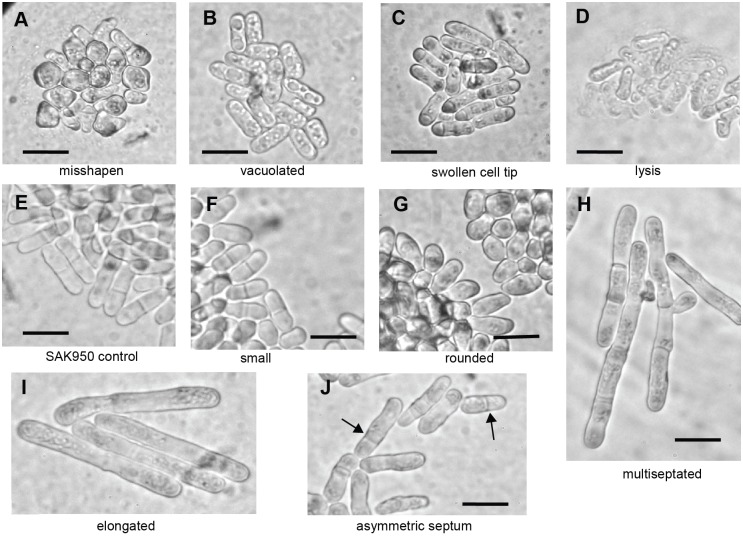
**Examples of *S. pombe* cells from plug assays that exhibit the different classes of cell shape defects.** Examples of (A) misshapen, (B) vacuolated, (C) swollen cell tip, (D) lysis, (E) control SAK950, (F) small, (G) rounded, (H) multiseptated, (I) elongated, (J) asymmetric septum cell shape defects. Black arrows in J indicate asymmetrically placed septa or an asymmetrically dividing cell. All cells within the halo, produced by the secreted bioactivity, were visually screened, and representative images from one screen are shown. Scale bars: 10 µm.

Microscopic examination of *S. pombe* cells showed that of the 420 actinomycete strains that produced a halo, 178 killed fission yeast without showing an obvious shape phenotype (described as ‘dead’ in Table S1). The remaining 242 strains produced activities that induced a range of altered *S. pombe* cell shape phenotypes. We classified these phenotypes into eight broad groups (Fig. 1, Tables S3 and S4), related to the phenotypes observed in the deletion mutant collection screen (Hayles et al., 2013). However, the range of phenotypes exhibited did not completely overlap; for example, analysis of the gene deletion collection did not identify the swollen cell tip phenotype shown in Fig. 1C, suggesting that bioactive compounds may induce a different range of phenotypes compared with the gene deletions. This may be, for example, because they can interact with several different target molecules/binding sites within the cell, selectively inhibit particular activities of multifunctional target molecule, or can modify, rather than inactivate, a target molecule. We found that several strains induced mixed phenotypes, for example, both ‘small’ and ‘rounded’, or both ‘asymmetric septum’ and ‘misshapen’; however, for the purposes of classification, each of the bacterial strains was allocated to a single phenotypic group based on its predominant phenotypic effect.

From the 242 strains with activities, we selected 149 strains, which included representatives of all phenotypes classes (Tables S1 and S3), for further investigation due to the high penetrance or interest of the induced phenotypes. From this subset of strains, we selected activities produced from B21P2 (asymmetric septum), T342 (elongated), 5307(1B) (swollen cell tip) and IS1 (rounded) actinomycete strains for immediate further analysis to establish whether our approach was useful.

### Characterization of an *S. pombe* asymmetry phenotype induced by B21P2

The asymmetric septation phenotype induced by B21P2 (Fig. 1J) was selected because this phenotype may be caused by a defect in spatial organisation within the cell. During interphase, microtubules are required for positioning of the nucleus in the middle of the cell, and the position of the septum at cytokinesis is normally dependent on the position of the nucleus (Chang and Nurse, 1996; Paoletti and Chang, 2000; Daga et al., 2006). It is therefore possible that this asymmetric septum phenotype induced by B21P2 is associated with other asymmetries and may be the result of a defective microtubule cytoskeleton and nuclear positioning.

**Fig. 2.**
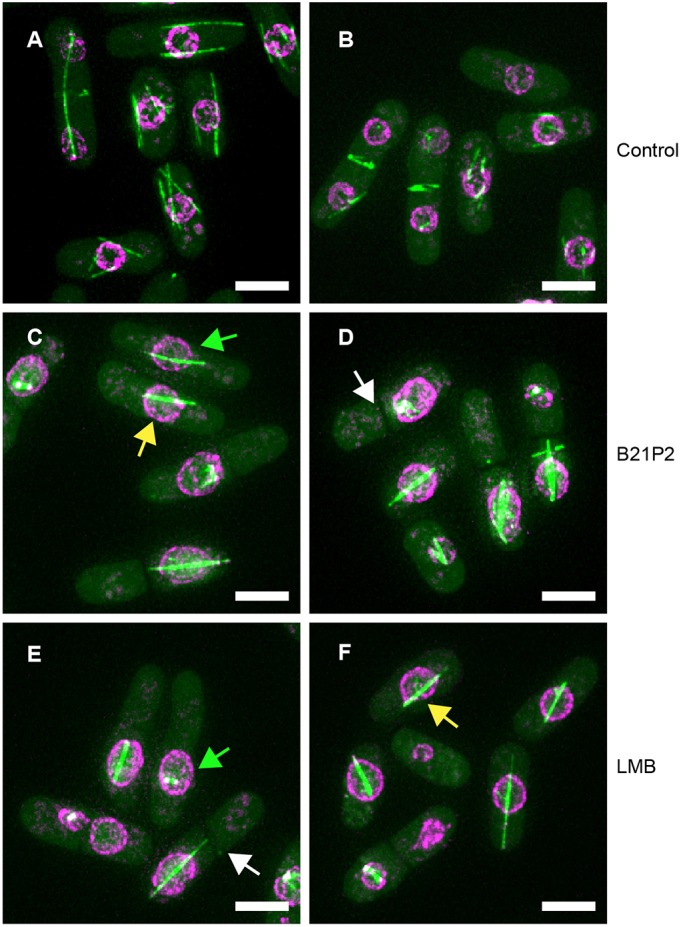
**Phenotype of *S. pombe* cells in the presence of B21P2 or LMB.** A total of 100 cells of strain PN5352 were examined for the major phenotypes of asymmetric nucleus (AN), extended spindle (ES) and asymmetric septum (AS) in the presence of B21P2 or LMB. Examples of *S. pombe* cells illustrating these defects are shown. (A,B) Cells in absence of drug (AN, *n*=0/100 cells; ES, *n*=0/100 cells; AS, *n*=0/100 cells). (C,D) Cells+B21P2 (1:100 dilution; AN, *n*=43/100 cells; ES, *n*=41/100 cells; AS, *n*=29/39 septated cells). (E,F) Cells+LMB (50 ng/ml; AN, *n*=30/100 cells; ES, *n*= 45/100 cells; AS, *n*=18/34 septated cells). Examples of asymmetric nucleus (green arrows), extended spindle (yellow arrows) and asymmetric septation (white arrows) are highlighted. Many cells show multiple defects. PN5352 cells were incubated in B21P2 or LMB for 2 h. Scale bars: 5 µm.

We treated PN5352 cells (Table S2) growing in YE4S liquid medium with semi-purified extracts from B21P2 to examine the microtubule cytoskeleton and nuclear position. A range of phenotypes, including reduction or absence of interphase microtubules, and asymmetric positioning of the nuclei and septum (Fig. 2, see legend for details; Movies 1 and 2) was observed. Cells also had a range of spindle defects, with one of the predominant phenotypes observed being a nuclear microtubule bundle or extended spindle (Fig. 2; Movie 2). It is possible that this microtubule bundle/spindle may be related to the intranuclear bundle observed when Ned1 is overproduced (Tange et al., 2002). Formation of both the mitotic spindle and interphase microtubules is dependent on the import and export of tubulin and factors required for polymerization of microtubules (Sato and Toda, 2010). This may be inhibited in the presence of B21P2 and lead to microtubule cytoskeletal defects. In addition to microtubule defects, we also observed asymmetric division of the nuclear membrane in the presence of an abnormal mitotic spindle. It has previously been observed that the nuclear membrane can undergo fission in the absence of a mitotic spindle (Castagnetti et al., 2010). Our results suggest that in the presence of B21P2 there may be a defect in spindle attachment to the nuclear membrane, which allows membrane fission to occur independently of spindle elongation (Movie 2, lower right cell). B21P2 may also directly affect the nuclear membrane and lead to differently sized nuclei (Fig. 2; Movie 2, upper right cell). Some of these microtubule and nuclear membrane defects have previously been described for cells treated with leptomycin B (LMB), which inhibits nuclear export (Nishi et al., 1994). We tested the effect of LMB on PN5352 cells, and found that the phenotype of SAK950 was almost identical to that seen with B21P2 (Fig. 2), suggesting the bioactive compound produced by this strain might be LMB.

### Purification and identification of the bioactive compound from B21P2

To identify the natural product produced by B21P2 that is responsible for the asymmetric septum phenotype, we purified it and compared it with LMB. We found that medium D (INA5) was the most effective production medium for the bioactive compound after 7 days growth. Using these conditions, agar plates were harvested and the bioactive compound was extracted by reverse-phase flash chromatography, followed by an ethyl acetate extraction, normal-phase flash chromatography, size exclusion chromatography, preparative high-performance liquid chromatography (HPLC) and mass spectrometry, as described in the Materials and Methods.

**Fig. 3.**
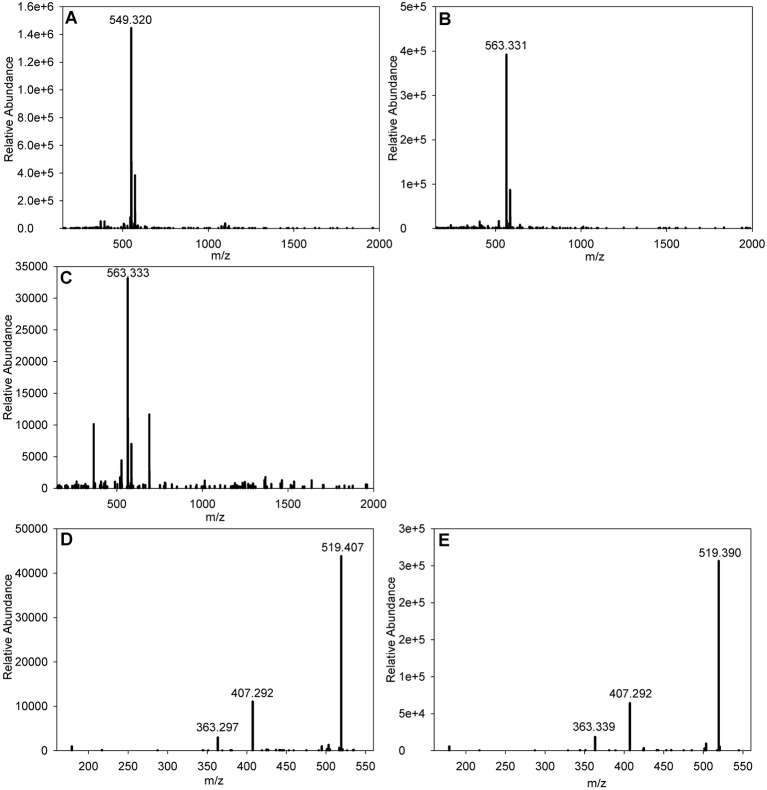
**Electrospray mass spectrometry data for LMA and LMB purified from B21P2 and commercially available LMB.** (A) B21P2 LMA [M+Na]^+^. (B) B21P2 LMB [M+Na]^+^. (C) Sigma LMB [M+Na]^+^. (D) MS/MS of the B21P2-derived LMB ion at *m*/*z*=563.331 shown in B. (E) MS/MS of Sigma LMB ion at *m*/*z*=563.333 shown in C.

The results of the purification indicated that there were two differing forms of the bioactive compound, which gave two discrete peaks in the HPLC elution at an λ=350 nm absorption spectrum trace. Eluate corresponding to both peaks was subjected to mass spectrometry, which revealed the presence of an ion at *m*/*z*=549.320 [M+Na]^+^ eluting at 14.6 min and an ion at *m*/*z*=563.331 [M+Na]^+^ eluting at 15.2 min (Fig. 3A,B shows the spectra from the two B21P2-derived active compounds). Querying of the ‘Dictionary of Natural Products’ (http://dnp.chemnetbase.com/faces/chemical/ChemicalSearch.xhtml) suggested that the most likely candidates were leptomycin A (LMA) and LMB (Hamamoto et al., 1983a,b). To confirm the identification of the purified B21P2 ion at *m*/*z*=563.331 [M+Na]^+^ as LMB, a sample of commercial LMB (Sigma L2913-5UG) was subjected to HPLC, where it eluted at 15.2 min, and mass spectrometry, where it gave an ion at *m*/*z*=563.333 [M+Na]^+^ (Fig. 3C). Additionally, the fragmentation pattern of the LMB-derived ion at *m*/*z*=563.331 [M+Na]^+^ (Fig. 3E) was identical to the fragmentation pattern of the B21P2-derived ion at *m*/*z*=563.333 [M+Na]^+^ (Fig. 3D) confirming that our initial identification of the B21P2 bioactive compound as LMB was correct.

### Elongated cell phenotype induced by T342

Next, we investigated activities causing an elongated cell phenotype (Fig. 1I): this phenotype is characteristic of a block in cell cycle progression while cells continue to grow (Bonatti et al., 1972) and screening for this phenotype has been instrumental in identifying many of the genes required for cell cycle progression (Nurse et al., 1976; Nurse and Thuriaux, 1980; Nasmyth and Nurse, 1981). One way in which the cell cycle could be blocked is by activation of a checkpoint delaying entry into mitosis in response to DNA damage or stalled replication forks (Enoch et al., 1992). Inability to activate the DNA checkpoint in the presence of DNA damage or stalled replication forks allows cells to enter mitosis to give a cut phenotype (Kelly et al., 1993; Saka and Yanagida, 1993). Cds1 is required to activate the intra-S checkpoint when DNA replication is stalled and Chk1 is activated in response to DNA damage. Rad3 is an upstream activator of both pathways (al-Khodairy et al., 1994). Actinomycetes are known to produce DNA-damaging compounds, for example, the anti-cancer agents bleomycin (Umenzawa et al., 1966) and adriamycin (Arcamone et al., 1969). We decided to focus on T342 as a representative example of an elongation-inducing strain and to investigate whether this phenotype was due to checkpoint activation by the bioactive natural product compound or a block in cell cycle progression in the absence of checkpoint activation.

### T342-induced cell elongation is Chk1 dependent

**Fig. 4.**
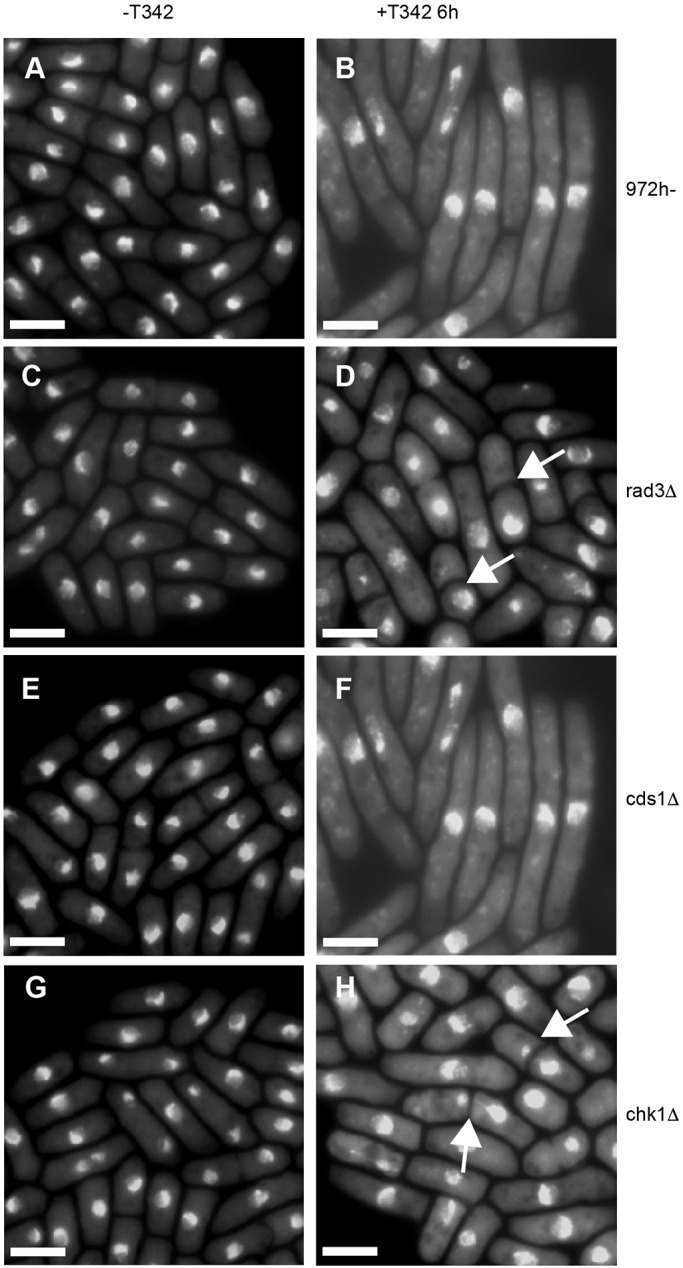
**T342 activates the DNA damage pathway.** (A) Wild-type 972h- cells in the absence of T342 or (B) 6 h after addition of T342. (C) *rad3Δ* cells in the absence of T342 or (D) 6 h after addition of T342. (E) *cds1Δ* cells in the absence of T342 or (F) 6 h after addition of T342. (G) *chk1Δ* cells in the absence of T342 or (H) 6 h after addition of T342. Cells were stained with DAPI to show DNA, and the white arrows show cells with a cut phenotype. Both 972 h- and *cds1Δ* cells elongated in the presence of T342; no cut cells were observed (*n*=200 cells). Neither *rad3Δ* nor *chk1Δ* cells showed any elongation (*n*=200 cells). For *chk1Δ*, 32% of cells and for *rad3Δ* 30% of cells showed abnormal DNA segregation (cut phenotype) (*n*=110 cells and *n*=100 cells, respectively). Scale bars: 10 µm.

When T342 extract was added to a Rad52–GFP-expressing strain (Table S2), the number of Rad52 foci increased compared to that found in cells in the absence of T342 suggesting that cell elongation in the presence of T342 is due to checkpoint activation as a result of either stalled DNA replication or DNA damage (Lisby et al., 2001) (Fig. S1). To test these two possibilities, we added semi-purified T342 extract to 972h- cells (T342 is active in both wild-type 972h- and SAK950 cells to a similar level; see Fig. S2, compare A with C), and *rad3Δ*, *chk1Δ* and *cds1Δ* strains (Table S2). Wild-type 972h- cells and cells lacking Cds1 elongated, suggesting that Cds1 (required for the intra-S checkpoint) is not required for checkpoint activation in the presence of T342 (Fig. 4A,B,E,F). Cells lacking Chk1 (required for the DNA damage checkpoint) or Rad3 (required upstream of both Cds1 and Chk1) did not elongate but entered mitosis prematurely to give a cut phenotype (Kelly et al., 1993; Saka and Yanagida, 1993; Yanagida, 1998) (Fig. 4B,C,G,H). These results demonstrate that the elongation phenotype was due to activation of the Chk1-dependent DNA checkpoint and suggest that T342 causes DNA damage.

### Purification and identification of the bioactive compound from T342

**Fig. 5.**
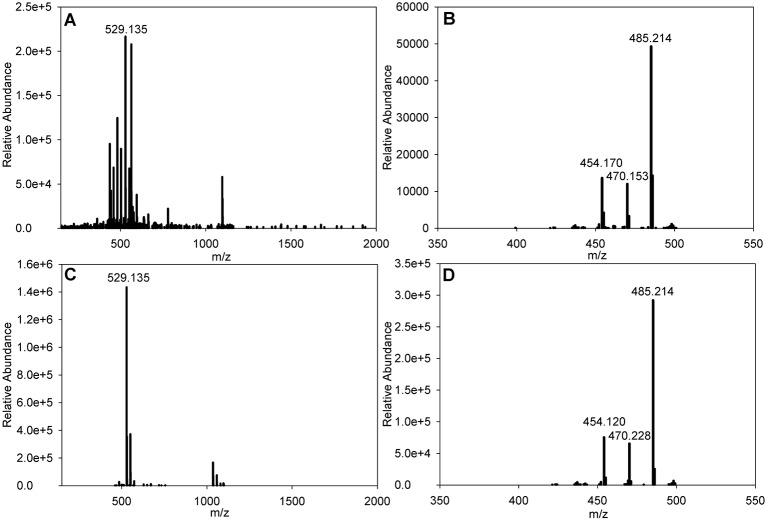
**Electrospray mass spectrometry data for SN purified from T342 and commercially available SN.** (A) SN purified from T342 [M+Na]^+^. (B) MS/MS of the T342-derived SN ion at *m*/*z*=529.135 shown in A. (C) Commercially available SN [M+Na]^+^. (D) MS/MS of the commercially available SN ion at *m*/*z*=529.135 shown in C.

Medium H (GYM) agar plates were inoculated with T342 and grown for 7 days, after which the plates were harvested and the bioactive compound was extracted in a multi-step procedure as described above. This initially comprised a ‘freebasing’ process followed by reverse-phase flash chromatography, preparative HPLC and mass spectrometry. The purification process resulted in identification of a bioactive compound, which eluted in the HPLC gradient at 11.5 min and produced an ion at *m*/*z*=529.135 [M+Na]^+^ (Fig. 5A). Querying of the Dictionary of Natural Products suggested that the most likely candidate was streptonigrin (SN), which possesses a very similar mass to that reported here. To confirm the identity of the purified T342 activity as SN, a sample of commercially purified SN (Sigma) was subjected to HPLC, where it eluted at 11.5 min, and mass spectrometry, where it gave an ion at *m*/*z*=529.135 [M+Na]^+^ (Fig. 5C). The fragmentation pattern of the ion at the *m*/*z*=529.1330 species [M+Na]^+^ generated by SN (Fig. 5D) was identical to the fragmentation pattern of the ion at *m*/*z*=529.1338 species [M+Na]^+^ from T342 (Fig. 5B) confirming that our initial identification was correct.

Additionally, we tested commercially available SN in a *S. pombe* filter paper assay to see whether this gave a similar phenotype to the purified bioactive compound from T342. We found that the phenotype using SN was indistinguishable from that seen with T342 (Fig. S2, compare C with D). Our results suggest that SN leads directly or indirectly to DNA damage, as previously reported (see Discussion).

### Swollen cell tip phenotype induced by 5307(1B)

**Fig. 6.**
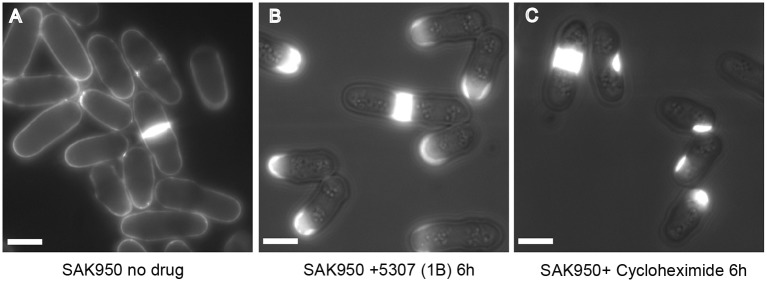
**SAK950 cells treated with 5307(1B) or commercially available cycloheximide.** Cells were stained with Calcofluor, a fluorescent stain that binds chitin and cellulose-like structures in the fungal cell wall and septum. (A) Untreated cells. (B) Cells after 6 h in 5307(1B) at 1:200 dilution of purified extract. Results are representative of *n*>100 cells; all cells showed abnormal Calcofluor staining. (C) Cells after 6 h in commercially available 1 mg/ml cycloheximide. Of *n*=145 cells, 82 cells showed abnormal Calcofluor staining and 63 cells showed no Calcofluor staining. Scale bars: 5 µm.

The phenotype induced by 5307(1B) was initially observed during the screen as a thickened region, mainly at one of the cell tips (Fig. 1C). Using a purer, more concentrated, extract we showed that the phenotype was due to accumulation of cell wall or septal material (Calcofluor staining material) at one of the cell tips (Fig. 6A,B) causing a swollen cell tip. At late time points, the cell tip became dramatically swollen (data not shown).

We purified the natural product produced by 5307(1B) that was responsible for this phenotype to conclusively identify the activity. We found that medium A was the most effective production medium for the bioactive compound after 7 days of growth. Using these conditions, agar plates were harvested and the bioactive compound was extracted by reverse-phase flash chromatography followed by an ethyl acetate extraction, normal-phase flash chromatography, preparative HPLC and mass spectrometry as described in the Materials and Methods.

**Fig. 7.**
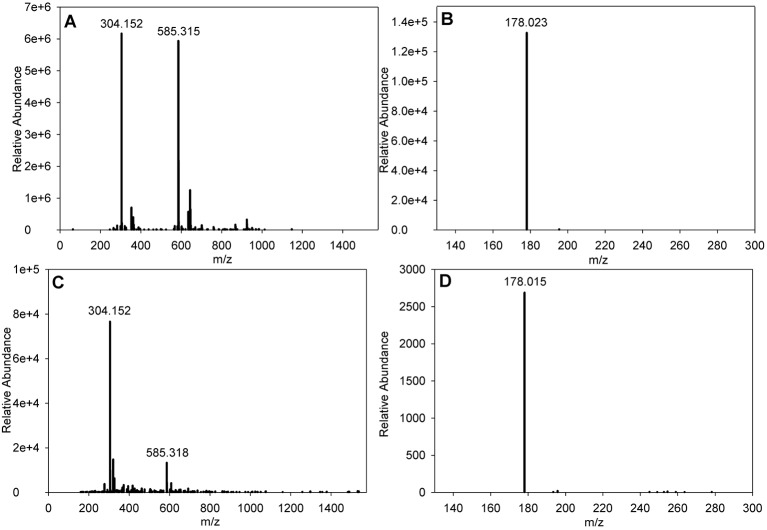
**Mass spectrometry data and fragmentation pattern for cycloheximide purified from 5307(1B) and commercial cycloheximide.** (A) 5307(1B) [M+Na]^+^. (B) MS/MS of the 5307(1B)-derived ion at *m*/*z*=304.152 shown in A. (C) Sigma cycloheximide [M+Na]^+^. (D) MS/MS of the Sigma cycloheximide-derived ion at *m*/*z*=304.152 shown in C.

Despite not being detected during the HPLC using any of the wavelengths used by the diode array detector (DAD) array, two fractions eluting at between 10 and 10.5 min containing the active compound were identified by filter disc bioassay. These were subjected to mass spectrometry and both revealed the presence of an ion at *m*/*z*=304.1525 [M+Na]^+^ (Fig. 7A). Querying of the Dictionary of Natural Products for a mass of 281.163 revealed cycloheximide to be a candidate, as this was given a result of 281.35. To test this, a sample of commercial cycloheximide (Melford Laboratories) was subjected to mass spectrometry where it gave an ion at *m*/*z*=304.152 [M+Na]^+^ (Fig. 7C). Additionally, the fragmentation pattern of the cycloheximide-derived ion at *m*/*z*=304.1519 [M+Na]^+^ was identical to the fragmentation pattern of the 5307(1B)-derived ion at *m*/*z*=304.1525 [M+Na]^+^ (Fig. 7B,D) confirming that our initial identification of the 5307(1B) bioactive compound as cycloheximide was correct.

To demonstrate that the phenotype exhibited by 5307(1B) and cycloheximide-treated *S. pombe* are comparable, cells were treated with 1 mg/ml cycloheximide (Melford Laboratories) and the phenotype was compared with that of 5307(1B) extract (1:200). In both cases, an accumulation of cell wall or septal material at or close to the cell tip and at the site of septation was observed (Fig. 6B,C). This phenotype has not been previously reported when *S. pombe* cells have been treated with cycloheximide. When a higher concentration of commercial cycloheximide (10 mg ml^−1^) was used with wild-type cells, SAK950 cells died and did not exhibit the cell wall phenotype.

### Rounded cell phenotype induced by IS1

**Fig. 8.**
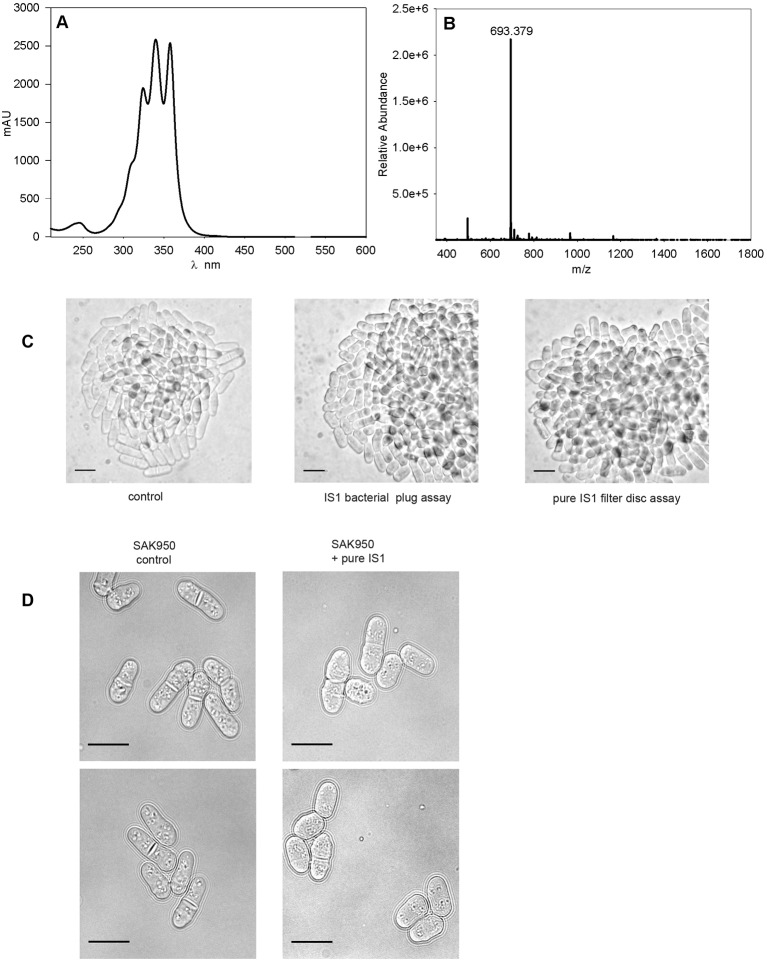
**The purified novel compound derived from IS1 causes a rounded cell phenotype.** (A) UV and visible absorbance spectrum of the IS1 compound. (B) Mass spectrometry data for compound purified from IS1 [M+H]^+^. (C) SAK950 control (left panel), SAK950 cells from the plug assay with IS1 (middle panel), and SAK950 cells from filter disc assays with ‘peak-picked’ purified IS1 compound (right panel). (D) Representative cells of SAK950 control (left panels) and SAK950+peak picked IS1 from B shown at higher magnification (right panels). Scale bars: 10 µm.

The final phenotype we investigated was the rounded cell phenotype (Fig. 1G) in the presence of IS1. This rounded category included phenotypes that varied from round cells to wider cells that were still rod-shaped. IS1 produced a mixed phenotype of rounded cells and more rod-shaped cells (Fig. 8C,D). Previous work has shown that rounded/wide cells can be the result of a number of different cellular defects. For example, defects in nutrient monitoring, growth zone size and assembly, actin delocalisation or cell wall defects can all generate this phenotype (Ribas et al., 1991; Mendoza et al., 2005; Leonhard and Nurse, 2005; Kelly and Nurse, 2011; Thompson and Sahai, 2015; Verde et al., 1995; Kume et al., 2013).

The compound produced by IS1, which was responsible for inducing the rounded cell phenotype, was successfully identified using the protocol described in the Materials and Methods. A peak common to the active fractions was identified, and a highly purified extract of the activity, which possesses a distinctive triple-peak absorbance pattern (Fig. 8A), was obtained by ‘peak picking’. A sample of this compound was subjected to mass spectrometry and revealed the presence of an ion at *m*/*z*=693.379 [M+H]^+^ (Fig. 8B). Querying of the Dictionary of Natural Products for an accurate mass of 692.371 did not produce any matches indicating, *prima facie*, that the compound represents a novel chemical entity.

To confirm that the activity purified from IS1 was responsible for the rounded cell phenotype, we carried out a filter paper assay against SAK950 cells and showed that a novel polyene induced the rounded cell phenotype (Fig. 8C,D). We identified IS1 as a polyene on the basis of the triple-peak absorbance pattern in the region of λ=300–450 nm, which is characteristic of polyenes and is used as a diagnostic aid to identify polyenes during natural product screening, see the Discussion for further details.

## DISCUSSION

In this study, we have screened 657 actinomyces strains for secretion of activities that affect the cell shape of fission yeast. From the set of 420 strains that produced bioactivities activities, there were 149 strains that we decided to follow up and here we have reported the identification by visual screening, and the purification and preliminary analysis, of four of these bioactivities as a proof of principle for this type of approach.

### Leptomycin origin and asymmetric phenotype bioactivity

The leptomycins were initially identified in 1983 (Hamamoto et al., 1983a,b), and the effect of LMB on *S. pombe* was studied soon after their purification (Hamamoto et al., 1985). It was shown to cause cell elongation, and an inhibitory effect of a high concentration of LMB (10 µg ml^−1^) on DNA synthesis was observed, although the primary effect was suggested to be inhibition of mitotic exit without blocking septation (Hamamoto et al., 1985). LMB-resistant mutants in *S. pombe* resulted in the identification of *crm1* as the cellular target and that LMB binds covalently to Crm1 at a cysteine residue in a conserved region of the protein (Kudo et al., 1998; Kudo et al., 1999). Crm1 mediates the export of proteins possessing nuclear export signals (NES) from the nucleus to the cytoplasm (Kudo et al., 1998; Fukuda et al., 1997; Stade et al., 1997) and has been implicated in a number of different cellular processes in different organisms, including centromere and telomere arrangement (Funabiki et al., 1993). Crm1 is linked to the spindle pole body (SPB) by Spc72 (Neuber et al., 2008) and is involved in locating pericentrin to the centrosome (Liu et al., 2009). In addition, it is associated with nucleophosmin and is involved in regulating centrosome duplication (Wang et al., 2005; Shinmura et al., 2005). It has also been shown that centrin and pericentrin accumulate in the nucleus following treatment with LMB (Keryer et al., 2003). LMB competes with the NES for stable formation of a complex with Ran-GTP. Ran-GTP stabilises microtubule plus-ends and has been shown to concentrate at the centrosome (Keryer et al., 2003), and in *S. pombe*, it interacts with at least one component of the SPB and controls microtubule integrity (Fleig et al., 2000).

Our results suggest that a significant effect of LMB is on the microtubule cytoskeleton, and we suggest that this leads directly to mis-positioning of the nucleus and septum. Although the LMB phenotype has been well characterised (see above), this is the first time that a septum-positioning defect has been reported for LMB in *S. pombe*. It is tempting to interpret the major LMB-induced *S. pombe* cell morphology phenotype in terms of disruption of Crm1 functions relating to the export of factors affecting the interphase microtubules, but it is possible that there are other factors whose Crm1-dependent export/localisation are also affected. Indeed, it has been shown that the localisation of 285 proteins, many of which are involved in cell cycle, cell division and cell morphology processes, are dependent on Crm1, and therefore subject to LMB-mediated disruption (Matsuyama et al., 2006).

Many tumour suppressors and oncoproteins use Crm1 for their nuclear export and, as the best-characterised nuclear export inhibitor, LMB has been a candidate for anti-cancer drug development, although it failed clinical trials due to toxicity. Nonetheless, the search for natural products that possess nuclear export inhibitory activity has continued (Cautain et al., 2014; Mutka, et al., 2009) and our results show that the *S. pombe* morphological effect could provide a means of screening for novel inhibitors of this class.

### SN origin and cell elongation phenotype

SN was first isolated from *Streptomyces flocculus* (Rao and Cullen, 1960), although identical molecules have also been isolated from other species; for example, ‘bruneomycin’ from *Actinomyces albus var bruneomycin* (Brazhnikova, et al., 1968). The structure of SN has been elucidated (Rao et al., 1963) and it possesses an aminoquinone moiety, in common with many other bioactive agents of actinomycete origin, including mitomycin C, actinomycin, rifamycin and geldanamycin. SN has been shown to exert its anti-tumour effects through direct and indirectly acting mechanisms (reviewed in Bolzán and Bianchi, 2001). SN acts to damage DNA directly when it forms complexes with metal ions, including Cu^2+^ (Sugiura et al., 1984), that are capable of intercalating with DNA (Cone et al., 1976). Also, when the quinone moiety undergoes autoxidation to semiquinone, in the presence of NADH, reactive oxygen species (ROS) capable of cleaving DNA are produced (Lown et al., 1978). However, SN also damages DNA indirectly through inhibition of topoisomerase II activity by binding to the minor groove and stabilising the transesterification intermediate of the enzyme, so inhibiting re-ligation of the DNA strands and generating DNA breaks (Yamashita et al., 1990; Capranico et al., 1994). It has also been shown through *in vitro* experiments that SN inhibits the ATPase activity of *S. cerevisiae* Rad54 by generating ROS and so prevents Holliday junction branch migration (Deakayne et al., 2013). *In vivo*, SN works through both the direct and indirect DNA-damaging mechanisms mentioned above, depending on its concentration, with the inhibition of topoisomerase function being the predominant mechanism at lower concentrations (Legault et al., 1997).

### Cycloheximide origin and cell wall defect phenotype

One of the more interesting aspects of the present study is the fact that in several cases the phenotypes observed during the screen are not due to a direct effect of the natural product compound on its cellular target but are due to downstream effects caused by inhibition of the primary target system. The cell wall defect phenotype observed to occur in response to 53017(1B) was initially expected to be due to an activity affecting cell wall biosynthesis, which led to a build-up of aberrant cell wall material within the cell and consequently to the observed distortions of cellular morphology. However, isolation of the active compound indicated that it was cycloheximide, whose identification was consistent with the similarity between the 16S rRNA gene sequence of strain 5307(1B) and that of *Streptomyces griseus*, which produces cycloheximide (actidione) (Whiffen et al., 1946; Leach et al., 1947; Kornfeld and Jones, 1948). Cycloheximide is well known as a general protein synthesis inhibitor (Kerridge, 1958) and is widely used as a research tool; therefore, the severe effects on the cell wall were unexpected. However, both cycloheximide and 5307(1B) extract induce a similar phenotype, with the *S. pombe* cell wall becoming thickened mainly at one of the cell tips. To our knowledge, this is the first time that an effect of cycloheximide on *S. pombe* cell morphology has been reported.

The differential effects of cycloheximide on the synthesis of the cell wall components mannan and glucan in *S. cerevisiae* have been noted previously, with mannan synthesis being inhibited, whereas glucan synthesis remains unaffected for a considerable time post cycloheximide treatment (Elorza, et al., 1976; Sentandreu and Lampen, 1970; Coen et al., 1994). The thickening of the *S. cerevisiae* cell wall following cycloheximide treatment has also been noted, particularly in the septum region at the tip of the bud (Rodriguez et al., 1979). Although the effect of cycloheximide treatment on *S. pombe* division has been investigated and the presence of thickened cell plates observed in dividing cells (Polanshek, 1977), the more-severe phenotype observed in this study has not been reported previously. Cycloheximide inhibition of protein synthesis in *S. pombe* may, as in *S. cerevisiae*, have a differential effect on mannan and glucan synthases. If so, then lack of an effect of cycloheximide on glucan synthases for a period of time after addition is likely to lead to the accumulation of unincorporated glucan at the septum in dividing cells and at the new cell tips generated at cell division, producing the aberrant cell wall *S. pombe* phenotype that we observed.

### Identification of the novel IS1 compound

The novel compound IS1 was identified through its ion at *m*/*z*=693.379 [M+H]^+^ by a search of the Dictionary of Natural Products. The determination of the absorbance spectrum of the molecules (Fig. 8A) indicated that it possesses a triple-peak pattern that is characteristic of molecules possessing a plurality of conjugated unsaturated carbon–carbon bonds (Oroshnik et al., 1955). Antifungal compounds that possess similar triple-peak absorbance spectra include the polyene macrolides (Oroshnik et al., 1955; Hamilton-Miller, 1973), and the triple-peak absorbance spectrum is widely used as a diagnostic tool in natural product screening to identify polyenes (Lemriss et al., 2003; Ouchdouch, et al., 2001). Based on the maxima of the absorption spectrum, we would predict that five or six unsaturated bonds are present (Hamilton-Miller, 1973) and, as during analysis of the IS1 compound by LC-MS a series of ions differing by 18 mass units were generated (data not shown), it is likely the IS1 compound possesses a multiplicity of hydroxyl groups that are lost via water as the major ion species fragments.

Additionally, the cell phenotype induced by IS1 is similar to but not identical to the rounded phenotype induced by the polyene antifungal nystatin (data not shown) and may reflect a similar mode of action. Sterols are well known to play an important role in maintaining the integrity of lipid rafts, which are responsible, at least in part, for the correct localisation of protein complexes necessary for cell morphology/polarity (Takeda, et al., 2004; Takeda and Chang, 2005; Wachtler et al., 2003; Codlin et al., 2008; Fischer et al., 2008). Although we do not attempt a precise explanation of the cell phenotype in terms of the action of the novel IS1 compound, or indeed of polyenes in general, we note that the rounded/wide phenotypes are consistent with reduced control of cell morphology/polarity which leads to loss of, or a less defined, rod-shaped phenotype.

The drug-sensitive strain used in this study, SAK950, possesses an *erg5Δ* mutation (Aoi et al., 2014) and so is deficient is ergosterol (Iwaki et al., 2008), which is known to be a target molecule of polyenes (Gray et al., 2012). Interestingly, although it was expected that SAK950 would therefore show some resistance to polyenes its sensitivity to nystatin is almost identical to the wild-type strain 972h-, although it is more sensitive than wild-type cells to other available antifungal agents (data not shown). Therefore, it seems that polyenes, in addition to targeting ergosterol itself, may also be able to exert a killing effect by interacting with the ergosterol precursors present in SAK950.

### Conclusions

Recently there has been a call for “more concerted screening campaigns of actinobacterial metabolites against model eukaryotes…. These screens should harness more than just live/dead screening. In other words, we should look for interesting developmental and behavioural phenotypes using well-developed model systems” (Ho and Nodwell, 2016). The screen described here aptly fits the above description; we have successfully screened strains producing putative anti-fungal natural products, from a major collection of actinomycete bacteria, for their ability to induce cell shape defects in *S. pombe*, with the aim of identifying chemical entities capable of exerting effects on the cell cycle/cell morphology. We were successful in identifying many such strains, which induced a wide range of aberrant phenotypes on *S. pombe*. The phenotypic groups we observed are similar to, but not completely overlapping with those observed after screening of the *S. pombe* genome-wide gene deletion collection (Hayles et al., 2013). As expected, the complexity of phenotypes we have identified suggests that bioactive compounds may induce a different range of phenotypic responses than a single gene knockout would generate, so demonstrating the richness and complexity of the biological activities the present study has identified.

Following on from the initial screen, we identified and purified four compounds, LMB, SN and cycloheximide produced by actinomycete bacteria B21P2, T342 and 5307(1B), respectively, and a novel compound from actinomycete bacterium IS1. These results demonstrate that this type of screen will identify both known compounds of biological interest and novel compounds. We have analysed the *S. pombe* phenotypes in the presence of these drugs and found that they correlate with the known or predicted bioactivities of these compounds. These four case studies confirm the validity of our approach to identify bioactivities affecting *S. pombe* cell cycle and cell morphology, and suggest that some of the other 149 activities that we are currently investigating will be novel, biologically important activities affecting these processes in *S. pombe*. Given the conservation of genes encoding proteins involved in the cell cycle and microtubule/actin cytoskeleton between *S. pombe* and higher eukaryotes, and the medical use of some of the compounds affecting the *S. pombe* cell wall, which may act as antifungals, it is likely that novel natural products identified in this way will also be relevant to higher eukaryotes.

## MATERIALS AND METHODS

### Yeast strains, growth conditions and media

Strains used in this study (Table S2) were grown at 30°C in YE4S (Moreno et al., 1991) unless otherwise stated.

### *S. pombe* morphological assays

#### Plug assay

1 ml pipette tips were used to cut agar plugs from lawns of bacterial strains on GYM agar plates (see below). A plug from each of three different bacterial strains plus an agar-only control plug was each placed in a quadrant on a YE4S plate seeded with 5×10^5^ mid-log phase SAK950 cells. Plates were incubated overnight at 30°C and screened the following day for the presence or absence of a halo in the *S. pombe* lawn surrounding the plugs and microscopically for the phenotype of the *S. pombe* cells (Fig. 1). This procedure was carried out after 5, 7 and 9 days of bacterial growth. As biomolecules diffuse through the agar at different rates and may have different effects at different concentrations, the phenotype of the cells was examined from the plug to the edge of the halo to ensure all phenotypes were observed (Table S1).

#### Filter disc assay

Filter discs soaked in extract (10–30 µl with an appropriate amount of H_2_O or solvent) (Whatman cat no. 2017-006) instead of bacterial plugs were used as described above.

### DNA damage and checkpoint activation

To assess DNA damage mediated by T342, the T342 extract was added to an exponentially growing culture of *rad52*GFP (Lisby et al., 2001) in YE4S liquid medium and the number of Rad52–GFP foci at 0, 1, 3 and 5 h after addition were counted and compared to the number upon no T342 extract addition.

To assess checkpoint activation mediated by T342, semi-purified T342 extract (1:100 dilution) was added to 972h-, *rad3Δ*, *cds1Δ* and *chk1Δ S. pombe* strains growing exponentially in YE4S medium. After 6 h, the cell phenotype was observed to see whether cells had elongated or had a cut phenotype (shown by DAPI staining).

### Microscopy

Bright-field images of live cells and DAPI-stained cell images were acquired on a Zeiss Axioskop 40 microscope with a Zeiss 63×/1.4 NA oil ph3 objective and a Zeiss Axiocam MRm camera. For the plug assay plates, cell images were captured with a Sony HD AVCHD camera using a Zeiss Akioskop 40 microscope with a 50×/0.56 NA Nikon objective and 2.5× Optovar magnification changer. PN5352 and Rad52–GFP live cells were imaged using a DeltaVision microscope (Applied Precision) and a 60×/1.42 NA oil immersion lens. A total of ten *z*-sections with 0.4 µm spacing were taken with a Coolsnap-HQ digital CCD camera (Roper Scientific) and deconvolved using SoftWoRx (Applied Precision). For time-lapse imaging, cells were grown in YE4S medium at 32°C to early exponential growth, B21P2 was added and cells were incubated for a further 40 min before transferring to the microfluidic cell culture system (CellASIC, ONIX). Six *z*-sections with 0.6 µm spacing were acquired for each time point with 5 min time intervals for 245 min at 32°C using the DeltaVision microscope (Applied Precision). For the SAK950 cells shown in Fig. 8D, live cells were imaged using a DeltaVision microscope (Applied Precision) with a 60×/1.42 NA oil immersion lens after resuspending cells from the edge of colonies in water.

Reagents were used at the following concentrations/dilutions: purified B21P2 at 1:100 dilution, LMB (Sigma L2913-5UG) at 50 ng/ml, partially purified T342 at 1:100, purified 5307(1B) at 1:200, xycloheximide (Melford Laboratories) at 1 mg/ml, Calcofluor (MD Biomedical) at 1:100 dilution of 1:1000 stock in PBS, and DAPI at 1 µg/ml. For filter disc assays, 10 µl aliquots of 20 µg/ml SN or undiluted T342 were used.

### Bacterial strains, growth conditions and media

The Demuris bacterial strain collection comprises over 10,000 actinomyces strains including a collection of marine-derived actinomycetes collected by Michael Goodfellow, Newcastle University, UK. The collection is notable for its diversity with many strains obtained from ecologically unexplored habitats and/or by novel isolation methodologies, and has been de-replicated to remove multiple identical strains. 657 strains previously shown to inhibit the growth of *S. cerevisae* and/or *S. pombe* were used in this study. This pre-screen used semi-purified extracts and/or plug assays for the presence or absence of a halo but the cell phenotype was not examined. We have examined all 657 strains over a longer time course for activities having an effect on cell shape in *S. pombe*. Strains were resuscitated from storage at −80°C on oatmeal agar plates (Kieser et al., 2000). For analysis, confluent bacterial lawns were streaked from the resuscitation plates on Medium H (GYM) agar plates (Kepplinger, 2016) and grown at 30°C. Growth media used to optimise production of the bioactivities were medium A, D (INA5), E (RA3), F (GPMY), G (V6), H (GYM), I, J, K and V (Kepplinger, 2016). All media were sterilised at 121°C for 10 min and solid media had agar added (10 g l^−1^). Strain B21P2 was isolated from the rhizosphere of *Paraserianthes falcataria* (Sembiring, 2000); the most closely related species is *Kitasatospora putterlickiae* (16S rRNA gene has 8/1386 bp mismatches). Strain T342 was first described in Sahin, 1995. The most closely related species is *Streptomyces albus* (16S rRNA gene has 14/1438 bp mismatches). The most closely related species to strain 5307(1B) (ATCC 12433) is *Streptomyces griseus* (16S rRNA gene has 0/1379 bp mismatches). The most closely related species to strain IS1 is *Streptomyces misionensis* (16S rRNA gene has 4/1414 bp mismatches).

### Production of water- and acetone-based bacterial cell extracts

B21P2 and IS1 were streaked onto GYM agar plates and grown for 7 days. Production of the bioactivity was assessed by using the plug assay. Agar from the plates was harvested, disrupted by passaging through a 50 ml syringe and frozen overnight at −20°C. A water-based extract was produced by squeezing the thawed slurry through muslin cheesecloth and filtering the resultant liquid through Whatman filter paper. A concentrated extract was obtained by freeze drying the filtered extract in an Edwards ‘Modulyo’ freeze dryer and resuspending the lyophilised material in an appropriate volume of water. The agar–cell mixture produced was resuspended in a volume of acetone equal to the initial volume of water-based extract and incubated overnight at 4°C. Acetone-based extract was produced by squeezing the mixture through muslin cheesecloth, filtering it and removal of acetone using a Büchi vacuum rotary evaporator to generate a concentrated extract comprising the residual water present in the spent agar–cell slurry.

### Production of bioactive crude extracts

B21P2 was streaked onto INA5 agar plates, T342 onto GYM agar plates and 5307(1B) onto Medium A plates. All strains were grown for 7 days and production of bioactive compounds assessed by use of the plug assay. Agar from the plates was harvested, disrupted by passaging through a 50 ml syringe and frozen overnight at −20°C. A water-based extract was produced by squeezing the thawed slurry through muslin cheesecloth and filtering the resultant liquid through Whatman filter paper. Washed Amberlite™ XAD16N resin beads (Sigma) were added (20 g l^−1^) to the resultant liquid to adsorb bioactive compounds. The liquid was successively extracted three times with portions of Amberlite™ resin beads, and the compounds released by washing the resin with volumes of methanol equal to the volume of the liquid extract. The methanol was removed by rotary evaporation (Büchi) to obtain water-based concentrates.

### Reverse-phase flash chromatography

The B21P2- and 5307(1B)-derived water-based concentrates, and IS1-derived acetone-based concentrates were passed through a pre-packed disposable reverse-phase C18 25 g SNAP flash chromatography column (Biotage) and the bound compounds eluted in a 0–100% water (0.1% formic acid)–methanol (0.1% formic acid) gradient using a Biotage Isolera One chromatography machine. T342-derived extract produced by resuspension of ‘freebased’ material (see ‘Solvent Extraction’ below) in water was treated similarly. For all strains, fractions containing bioactive compounds were identified by spotting 30 µl aliquots onto filter paper discs, which were used in the filter disc assay. Active fractions producing a halo of cells with the expected characteristic phenotypes were pooled and the methanol removed by a GeneVac Series II system equipped with a GeneVac VC3000TA condenser unit.

### Thin layer chromatography

B21P2- and 5307(1B)-derived samples were subjected to thin layer chromatography (TLC) using silica gel plates (Fluka Analytical) and a range of solvents: ethyl acetate, methanol and diethyl ether. After running the samples, the TLC plates were pressed, silica side down, onto YE4S agar plates and the position of the TLC plates, sample loading site and solvent front were recorded on the back of the petri dish. After a 30 min transfer period the TLC plates were removed and the YE4S agar plates were inoculated with *S. pombe* in a similar fashion to in the plug and filter paper assays. After an overnight incubation at 30°C, zones of inhibition in the *S*. *pombe* lawns comprising cells of aberrant morphology indicated the presence of bioactive compounds of interest and comparison of the location of these zones with the solvent front and sample loading site markings allowed assessment of the solubility of the bioactive compound in the various solvents and selection of the most appropriate solvent(s) for future purification steps.

### Solvent extraction

Ethyl acetate extractions of B21P2- and 5307(1B)-derived material were obtained by shaking the aqueous extract with an equal volume of ethyl acetate in a separating funnel for 5 min followed by separation of the two phases. The ethyl acetate phase was rotary evaporated with silica so as to adsorb the compounds therein. A ‘freebasing’ process was employed with regard to T342-derived extracts where the pH of the aqueous extract (pH 8) was raised to pH 10 and an ethyl acetate extraction used to remove contaminants in the organic phase. This was followed by alteration of the pH of the aqueous phase to pH 4 and again obtaining an ethyl acetate extraction where the bioactive compound migrated into the organic phase, after which it was recovered by rotary evaporation (Büchi) and resuspended in water.

### Normal-phase flash chromatography and size exclusion chromatography

The silica with adsorbed B21P2-derived compound was transferred into a pre-packed, disposable K-Sil 25 g SNAP column (Biotage) and used in normal-phase flash chromatography with elution in a 0–100% diethyl ether–ethyl acetate gradient. The silica with adsorbed 5307(1B)-derived compound was similarly treated with elution in a 0–100% dichloromethane–methanol gradient. For both strains, fractions containing bioactive compounds were identified by spotting 30-µl aliquots onto filter paper discs which were used, as described above, to identify active fractions producing a halo comprising cells exhibiting the expected characteristic phenotypes. For B21P2, active fractions were pooled, the solvent removed using a GeneVac Series II and the resultant sample was loaded onto a 150 PSI Max (Omnifit) size exclusion column packed with Sephadex™ LH-20 connected to a Biotage Isolera One chromatography machine and eluted in a methanol mobile phase. Active fractions, identified by the filter paper assay, were pooled and the methanol removed using a GeneVac Series II.

### HPLC and mass spectrometry

The B21P2-derived compound was solubilised by addition of 25% methanol, and 100 µl aliquots were passed through a Perkin Elmer Series 200 HPLC machine equipped with a Phenomenex 150×4.6 mm Synergy 4 µm reverse-phase column and a Hichrom C18 guard column and eluted using a 43 min 25%–100% water (0.1% formic acid)–acetonitrile (0.1% formic acid) gradient, with a flow rate of 1 ml min^−1^ and monitoring at λ=254 nm. Fractions (0.5 ml) were collected in a 96-well block, and active fractions identified by the filter paper assay. Following correlation of peaks in the UV absorbance trace with the active fractions, the method was repeated with an Agilent Technologies 1260 Infinity liquid chromatography machine equipped with an integrated fraction collector so as to ‘peak pick’ and collect column eluate corresponding to the peaks of interest. The gradient used was 25%–100% water (0.1% formic acid)–acetonitrile (0.1% formic acid) over 15 min ending with a 5 min 100% acetonitrile column wash using a flow rate of 1 ml min^−1^ and monitoring with a DAD array at λ=210, 254, 273, 280, 300, 350 and 600 nm relative to λ=360 nm. T342- and 5307(1B)-derived compounds were similarly analysed but were eluted in a gradient of 15%–100% water (0.1% formic acid)–acetonitrile (0.1% formic acid). IS1-derived compound was passed through an Agilent 150×4.6 mm Eclipse Plus C18 3.5 µm reverse-phase column and a Hichrom C18 guard column and eluted using a 30 min 30%–100% water– acetonitrile gradient, with a flow rate of 1 ml min^−1^ and monitoring at λ=350 nm with an Agilent Technologies 1260 Infinity liquid chromatography machine equipped with an integrated fraction collector so as to peak pick and collect column eluate corresponding to the peak of interest.

After removal of solvent, by using the GeneVac Series II, and addition of 25% methanol to enhance solubility, the samples were analysed by electrospray mass spectrometry (ESI-MS) using a LTQ-FT (Thermo) mass spectrometer with a 7T magnet at the Pinnacle Laboratory (University of Newcastle). Experiments were run with a parent/precursor scan at 100,000 resolution. MS/MS fragmentations were carried out in the ion-trap (LTQ) stage of the instrument. Commercially available LMB (Sigma), SN (Sigma) and cycloheximide (Melford Laboratories) were used as comparative standards for the purified molecules of interest. For HPLC, LMB (6.5 µg/ml) and SN (20 µg/ml) were made up in 20% methanol, and for mass spectrometry, LMB (11 µg ml^−1^) was in 50% methanol whereas SN (20 µg ml^−1^) and cycloheximide (10 µg ml^−1^) were in 20% methanol.

## References

[JCS194571C1] al-Khodairy F., Fotou E., Sheldrick K. S., Griffiths D. J., Lehmann A. R. and Carr A. M. (1994). Identification and characterization of new elements involved in checkpoint and feedback controls in fission yeast. *Mol. Biol. Cell* 5, 147-160. 10.1091/mbc.5.2.147 8019001 PMC301021

[JCS194571C2] Aoi Y., Sato M., Sutani T., Shirahige K., Kapoor T. M. and Kawashima S. A. (2014). Dissecting the first and second meiotic divisions using a marker-less drug-hypersensitive fission yeast. *Cell Cycle* 13, 1327-1334. 10.4161/cc.28294 24621506 PMC4049969

[JCS194571C3] Arcamone F., Cassinelli G., Fantini G., Grein A., Orezzi P., Pol C. and Spalla C. (1969). Adriamycin, 14-hydroxydaunomycin, a new antitumor antibiotic from *S. peucetius var. caesius*. *Biotechnol. Bioeng.* 11, 1101-1110. 10.1002/bit.260110607 5365804

[JCS194571C4] Beauchamp E. M. and Platanias L. C. (2013). The evolution of the TOR pathway and its role in cancer. *Oncogene* 32, 3923-3932. 10.1038/onc.2012.567 23246968

[JCS194571C5] Bolzán A. D. and Bianchi M. S. (2001). Genotoxicity of Streptonigrin: a review. *Mutat. Res.* 488, 25-37. 10.1016/S1383-5742(00)00062-4 11223403

[JCS194571C6] Bonatti S., Simili M. and Abbondandola A. (1972). Isolation of temperature sensitive mutants of *Schizosaccharomyces pombe*. *J. Bact.* 109, 484-491. 4110142 10.1128/jb.109.2.484-491.1972 PMC285166

[JCS194571C7] Brazhnikova M. G., Ponomarenko V. I., Kovsharova I. N., Kruglyak E. B. and Proshlyakova V. V. (1968). Study on bruneomycin produced by *Act. Albus var. bruneomycini* and its identification with Streptonigrin. *Antibiotiki* 13, 99-102. 4970063

[JCS194571C8] Broustas C. G. and Lieberman H. B. (2014). DNA damage response genes and the development of cancer metastasis. *Radiat. Res.* 181, 111-130. 10.1667/RR13515.1 24397478 PMC4064942

[JCS194571C9] Capranico G., Palumbo M., Tinelli S. and Zunio F. (1994). Unique sequence specificity of topoisomerase II DNA cleavage stimulation and DNA binding mode of Streptonigrin. *J. Biol. Chem.* 269, 25004-25009. 7929186

[JCS194571C11] Castagnetti S., Oliferenko S. and Nurse P. (2010). Fission yeast cells undergo nuclear division in the absence of spindle microtubules. *PLoS Biol.* 8, e1000512 10.1371/journal.pbio.1000512 20967237 PMC2953530

[JCS194571C12] Cautain B., de Pedro N., Garzon V. M., de Escalona M. M., Menedez V. G., Tormo J. R., Martin J., El Aoud N., Reyes F., Asensio F. et al (2014). High-content screening of natural products reveals novel nuclear export inhibitors. *J. Biomol. Screen* 91, 57-65. 10.1177/1087057113501389 24045581

[JCS194571C13] Chang F. and Nurse P. (1996). How fission yeast fission in the middle. *Cell* 84, 191-194. 10.1016/S0092-8674(00)80973-3 8565064

[JCS194571C14] Codlin S., Haines R. L. and Mole S. E. (2008). *btn1* affects endocytosis, polarization of sterol-rich membrane domains and polarized growth in *Schizosaccharomyces pombe*. *Traffic* 9, 936-950. 10.1111/j.1600-0854.2008.00735.x 18346214 PMC2440566

[JCS194571C15] Coen M. L., Lerner C. G., Capobianco J. O. and Goldman R. C. (1994). Synthesis of yeast cell wall glucan and evidence for glucan metabolism in a *Saccharomyces cerevisiae* whole cell system. *Microbiology* 140, 2229-2237. 10.1099/13500872-140-9-2229 7952174

[JCS194571C16] Cone R., Hasan S. K., Lown J. W. and Morgan A. R. (1976). The mechanism of degradation of DNA by Streptonigrin. *Can. J. Biochem.* 54, 219-223. 10.1139/o76-034 1260505

[JCS194571C17] Daga R. R., Yonetani A. and Chang F. (2006). Asymmetric microtubule pushing forces in nuclear centering. *Curr. Biol.* 16, 1544-1550. 10.1016/j.cub.2006.06.026 16890530

[JCS194571C18] Deakayne J. S., Huang F., Negri J., Tolliday N., Cocklin S. and Mazin A. V. (2013). Analysis of the activities of RAD54, a SWI2/SNF protein, using a specific small-molecule inhibitor. *J. Biol. Chem.* 288, 31567-31580. 10.1074/jbc.M113.502195 24043618 PMC3814753

[JCS194571C19] Dekanty A., Barrio L. and Milán M. (2015). Contributions of DNA repair, cell cycle checkpoints and cell death to suppressing the DNA damage-induced tumorigenic behavior of *Drosophila* epithelial cells. *Oncogene* 34, 978-985. 10.1038/onc.2014.42 24632609

[JCS194571C20] Deshpande G. F., Hayles J., Hoe K. L., Kim D. U., Park H. O. and Hartsuiker E. (2009). Screening a genome-wide *S. pombe* deletion library identifies novel genes and pathways involved in genome stability maintenance. *DNA Repair (Amst)* 8, 672-679. 10.1016/j.dnarep.2009.01.016 19264558 PMC2675035

[JCS194571C21] Elorza M. V., Lostau C. M., Villanueva J. R. and Sentandreu R. (1976). Cell wall synthesis regulation in *Saccharomyces cerevisiae*. Effect on RNA and protein inhibition. *Biochimica et Biophysica Acta.* 454, 263-272. 10.1016/0005-2787(76)90229-X 793624

[JCS194571C22] Enoch T., Carr A. M. and Nurse P. (1992). Fission yeast genes involved in coupling mitosis to completion of DNA replication. *Genes Dev.* 6, 2035-2046. 10.1101/gad.6.11.2035 1427071

[JCS194571C23] Fischer R., Zekert N. and Takeshita N. (2008). Polarized growth in fungi - interplay between the cytoskeleton, positional markers and membrane domains. *Mol. Microbiol.* 68, 813-826. 10.1111/j.1365-2958.2008.06193.x 18399939

[JCS194571C24] Fleig U., Salus S., Karig I. and Sazer S. (2000). The fission yeast ran GTPase is required for microtubule integrity. *J. Cell. Biol.* 151, 1101-1111. 10.1083/jcb.151.5.1101 11086011 PMC2174346

[JCS194571C25] Fukuda M., Saano S., Nakamura T., Adachi M., Yoshida M., Yanagida M. and Nishida E. (1997). CRM1 is responsible for intracellular transport mediated by the nuclear export signal. *Nature* 390, 308-311. 10.1038/36894 9384386

[JCS194571C26] Funabiki H., Hagan I., Uzawa S. and Yanagida M. (1993). Cell cycle-dependent specific positioning and clustering of centromeres and telomeres in fission yeast. *J. Cell. Biol.* 121, 961-976. 10.1083/jcb.121.5.961 8388878 PMC2119680

[JCS194571C27] Gatti L., Hoe K. L., Hayles J., Righetti S. C., Carenini N., Bo L. D., Kim D. U., Park H. O. and Perego P. (2011). Ubiquitin- proteosome genes as targets for modulation of cisplatin sensitivity in fission yeast. *BMC Genomics* 12, 44 10.1186/1471-2164-12-44 21247416 PMC3032702

[JCS194571C28] Gould K. L. and Nurse P. (1989). Tyrosine phosphorylation of the fission yeast cdc2+ protein kinase regulates entry into mitosis. *Nature* 342, 39-45. 10.1038/342039a0 2682257

[JCS194571C29] Graml V., Studera X., Lawson J. L. D., Chessel A., Geymonat M., Bortfeld-Miller M., Walter T., Wagstaff L., Piddini E. and Carazo-Salas R. E. (2014). A genomic Multiprocess survey of machineries that control and link cell shape, microtubule organization and cell-cycle progression. *Dev. Cell* 31, 227-239. 10.1016/j.devcel.2014.09.005 25373780 PMC4648281

[JCS194571C30] Gray K. C., Palacios D. S., Dailey I., Endo M. M., Uno B. E., Wilcock B. C. and Burke M. D. (2012). Amphotericin primarily kills yeast by simply binding ergosterol. *Proc. Natl. Acad. Sci. USA* 109, 2234-2239. 10.1073/pnas.1117280109 22308411 PMC3289339

[JCS194571C32] Hamamoto T., Gunji S., Tsuji H. and Beppu T. (1983a). Leptomycins A and B, new antifungal antibiotics I taxonomy of the producing strain and their fermentation, purification and characteristics. *J. Antibiot.* 36, 639-645. 10.7164/antibiotics.36.639 6874585

[JCS194571C33] Hamamoto T., Seto H. and Beppu T. (1983b). Leptomycins A and &, new antifungal antibiotics II structure elucidation. *J. Antibiot.* 36, 646-650. 10.7164/antibiotics.36.646 6874586

[JCS194571C34] Hamamoto T., Uozumi T. and Beppu T. (1985). Leptomycins A and &, new antifungal antibiotics III mode of action of leptomycin B on *Schizosaccharomyces pombe*. *J. Antibiot.* 38, 1573-1580. 10.7164/antibiotics.38.1573 4077736

[JCS194571C35] Hamilton-Miller J. M. T. (1973). Chemistry and biology of the polyene macrolide antibiotics. *Bacteriol. Rev.* 37, 166-196. PMC413825 4202146

[JCS194571C36] Hayles J., Wood V., Jeffery L., How K.-L., Kim D.-U., Park H.-O., Salas-Pino S., Heichinger C. and Nurse P. (2013). A genome wide resource of cell cycle and cell shape genes of fission yeast. *Open Biol.* 3, 130053 10.1098/rsob.130053 23697806 PMC3866870

[JCS194571C37] Heisler J., Elvir L., Barnouti F., Charles E., Wolkow T. D. and Pyati R. (2014). Morphological effects of natural products on *Schizosaccharomyces pombe* measured by imaging flow cytometry. *Nat. Prod. Bioprospect.* 4, 27-35. 10.1007/s13659-014-0004-8 24660134 PMC3956978

[JCS194571C38] Ho L. K. and Nodwell J. R. (2016). David and Goliath: chemical perturbation of eukaryotes by bacteria. *J. Ind. Microbiol. Biotechnol.* 43, 233-248. 10.1007/s10295-015-1686-6 26433385 PMC4752587

[JCS194571C39] Ingber D., Fujita T., Kishimoto S., Sudo K., Kanamura T., Brem H. and Folkman J. (1990). Synthetic analogues of Fumagillin that inhibit angiogenesis and suppress tumour growth. *Nature* 348, 555-557. 10.1038/348555a0 1701033

[JCS194571C40] Iwaki T., Iefuji H., Hiraga Y., Hosomi A., Morita T., Giga-Hama Y. and Takegawa K. (2008). Multiple functions of ergosterol in the fission yeast *Schizosaccharomyces pombe*. *Microbiology* 154, 830-841. 10.1099/mic.0.2007/011155-0 18310029

[JCS194571C41] Johnson R. and Halder G. (2014). The two faces of Hippo: targeting the Hippo pathway for regenerative medicine and cancer treatment. *Nat. Rev. Drug Discov.* 13, 63-79. 10.1038/nrd4161 24336504 PMC4167640

[JCS194571C42] Kawashima S. A., Chen Z., Aoi Y., Patgiri A., Kobayashi Y., Nurse P. and Kapoor T. M. (2016). Potent, reversible, and specific chemical inhibitors of eukaryotic ribosome biogenesis. *Cell* 167, 512-524. 27667686 10.1016/j.cell.2016.08.070 PMC5116814

[JCS194571C43] Kelly F. D. and Nurse P. (2011). Spatial control of Cdc42 activation determines cell width in fission yeast. *Mol. Biol. Cell.* 22, 3801-3811. 10.1091/mbc.E11-01-0057 21849474 PMC3192860

[JCS194571C44] Kelly T. J., Martin G. S., Forsburg S. L., Stephen R. J., Russo A. and Nurse P. (1993). The fission yeast cdc18+ gene product couples S phase to START and mitosis. *Cell* 74, 371-382. 10.1016/0092-8674(93)90427-R 7916658

[JCS194571C45] Kepplinger B. (2016). The discovery and characterisation of antibiotics from *Amycolatopsis* isolate DEM30355. *EngD thesis*, University of Newcastle, Newcastle-upon Tyne, UK.

[JCS194571C46] Kerridge D. (1958). The effect of actidione and other antifungal agents on nucleic acid and protein synthesis in *Saccharomyces carlsbergensis**.* *J. Gen. Microbiol.* 19, 497-506. 10.1099/00221287-19-3-497 13611192

[JCS194571C47] Keryer G., DiFiore B., Celati C., Lechtreck K. F., Mogensen M., Delouvée A., Lavia P., Bornens M. and Tassin A.-M. (2003). Part of Ran is associated with AKAP450 at the centrosome: involvement in microtubule –organising activity. *Mol. Biol. Cell.* 14, 4260-4271. 10.1091/mbc.E02-11-0773 14517334 PMC207017

[JCS194571C48] Kieser T., Bibb M. J., Buttner M. J., Chater K. F. and Hopwood D. A. (2000). *Practical Streptomyces Genetics*. Norwich: The John Innes Foundation.

[JCS194571C50] Kornfeld E. C. and Jones R. G. (1948). The structure of actidione, and antibiotic from *Streptomyces griseus*. *Science* 108, 437-438. 10.1126/science.108.2808.437-a 17736575

[JCS194571C51] Kudo N., Wolff B., Sekimoto T., Schreiner E. P., Yoneda Y., Yanagida M., Hornouchi S. and Yoshida M. (1998). Leptomycin B inhibition of signal mediated nuclear export by direct binding to Crm1. *Exp. Cell. Res.* 242, 540-547. 10.1006/excr.1998.4136 9683540

[JCS194571C52] Kudo N., Matsumori N., Taoka H., Fujiwara D., Schrreiner E. P., Wolff B., Yoshida M. and Horinouch S. (1999). Leptomycin B inactivates CRM1/exportin by covalent modification at a cysteine residue in the central conserved region. *Proc. Natl. Acad. Sci. USA* 96, 9112-9117. 10.1073/pnas.96.16.9112 10430904 PMC17741

[JCS194571C53] Kume K., Kubota S., Koyano T., Kanai M., Mizunuma M., Toda T. and Hirata D. (2013). Fission yeast leucine-rich repeat protein Lrp1 is essential for cell morphogenesis as a component of the morphogenesis Orb6 network (MOR). *Biosci. Biotechnol. Biochem.* 77, 1086-1091. 10.1271/bbb.130064 23649273

[JCS194571C54] Leach B. E., Ford J. H. and Whiffen A. J. (1947). Actidione, an antibiotic from *Streptomyces griseus*. *J. Am. Chem. Soc.* 69, 474 10.1021/ja01194a519 20292455

[JCS194571C55] Legault J., Tremblay A., Ramotar D. and Mirault M.-E. (1997). Clusters of S1 nuclease-hypersensitive sites induced in vivo by DNA damage. *Mol. Cell. Biol.* 17, 5437-5452. 10.1128/MCB.17.9.5437 9271420 PMC232393

[JCS194571C56] Lemriss S., Laurent F., Couble A., Casoli E., Lancelin J. M., Saintpierre-Bonaccio D., Rifai S., Fassouane A. and Boiron P. (2003). Screening of nonpolyenic antifungal metabolites produced by clinical isolates of actinomycetes. *Can. J. Microbiol.* 49, 669-674. 10.1139/w03-088 14735216

[JCS194571C57] Leonhard K. and Nurse P. (2005). Ste20/GCK kinase Nak1/Orb3 polarizes the actin cytoskeleton in fission yeast during the cell cycle. *J. Cell. Sci.* 118, 1033-1044. 10.1242/jcs.01690 15731009

[JCS194571C60] Lisby M., Rothstein R. and Mortensen U. H. (2001). Rad52 forms DNA repair and recombination centers during S phase. *Proc. Natl. Acad. Sci. USA* 98, 8276-8282. 10.1073/pnas.121006298 11459964 PMC37432

[JCS194571C61] Liu Q., Jiang Q. and Zhang C. (2009). A fraction of Crm1 locates at centrosomes by its CRIME domain and regulates the centrosomal location of pericentrin. *Biochem. Biophys. Res. Commun.* 384, 383-388. 10.1016/j.bbrc.2009.04.154 19422798

[JCS194571C62] Lown J. W., Sim S.-K. and Chen H.-H. (1978). Hydroxyl radical production by free and bound aminoquinone antibiotics. *Can. J. Biochem.* 56, 1042-1047. 10.1139/o78-164 216472

[JCS194571C63] Matsuyama A., Arai R., Yashiroda Y., Shirai A., Kamata A., Sekido S., Kobayashi Y., Hashimoto A., Hamamoto M., Hiraoka Y. et al (2006). ORFeome cloning and global analysis of protein localization in the fission yeast *Schizosaccharomyces pombe*. *Nat. Biotechnol.* 27, 841-847. 10.1038/nbt1222 16823372

[JCS194571C64] Mendoza M., Redemann S. and Brunner D. (2005). The fission yeast MO25 protein functions in polar growth and cell separation. *Eur. J. Cell. Biol.* 84, 915-926. 10.1016/j.ejcb.2005.09.013 16325501

[JCS194571C65] Moreno S., Klar A. and Nurse P. (1991). Molecular genetic analysis of fission yeast schizosaccharomyces pombe. *Methods Enzymol.* 194, 795-823. 10.1016/0076-6879(91)94059-L 2005825

[JCS194571C66] Mitchison J. M. and Nurse P. (1985). Growth in cell length in the fission yeast *Schizosaccharomyces pombe*. *J. Cell Sci.* 75, 357-376. 4044680 10.1242/jcs.75.1.357

[JCS194571C67] Mutka S. C., Yang W. Q., Dong S. D., Ward S. L., Craig D. A., Timmermans P. B. and Murli S. (2009). Identification of the nuclear export inhibitors with potent anticancer activity *in vivo*. *Cancer Res.* 69, 510-517. 10.1158/0008-5472.CAN-08-0858 19147564 PMC2635062

[JCS194571C68] Nasmyth K. and Nurse P. (1981). Cell division cycle mutants altered in DNA replication and mitosis in the fission yeast *Schizosaccharomyces pombe*. *Mol. Gen. Genet.* 182, 119-124. 10.1007/BF00422777 6943408

[JCS194571C69] Navarro F. J. and Nurse P. (2012). A systematic screen reveals new elements acting at the G2/M cell cycle control. *Genome Biol.* 13, R36 10.1186/gb-2012-13-5-r36 22624651 PMC3446289

[JCS194571C70] Neuber A., Franke J., Wittstruck A., Schlenstedt G., Sommer T. and Stade K. (2008). Nuclear export receptor Xpo1/Crm1 is physically and functionally linked to the spindle pole body in budding yeast. *Mol. Cell Biol.* 28, 5348-5358. 10.1128/MCB.02043-07 18573877 PMC2519715

[JCS194571C71] Nishi K., Yoshida M., Fujiwara D., Nishiikawa M., Horinouchi S. and Beppu T. (1994). Leptomycin B targets a regulatory cascade of crm1, a fission yeast nuclear protein, involved in control of higher order chromosome structure and gene expression. *J. Biol. Chem.* 269, 6320-6324. 8119981

[JCS194571C72] Nurse P. (1990). Universal control mechanism regulating onset of M-phase. *Nature* 344, 503-508. 10.1038/344503a0 2138713

[JCS194571C73] Nurse P. and Thuriaux P. (1980). Regulatory genes controlling mitosis in the fission yeast *Schizosaccharomyces pombe*. *Genetics* 96, 627-637. 7262540 10.1093/genetics/96.3.627 PMC1214365

[JCS194571C74] Nurse P., Thuriaux P. and Nasmyth K. (1976). Genetic control of the cell division in the fission yeast *Schizosaccharomyces pombe**.* *Mol. Gen. Genet.* 146, 167-178. 10.1007/BF00268085 958201

[JCS194571C75] Oroshnik W., Vining L. C., Mebane A. D. and Taber W. A. (1955). Polyene antibiotics. *Science* 121, 147-149. 10.1126/science.121.3136.147 13225759

[JCS194571C76] Ouchdouch Y., Barakate M. and Finance C. (2001). Actinomycetes of Moroccan habitats: isolation and screening for antifungal activities. *Eur. J. Soil Biol.* 37, 69-74. 10.1016/S1164-5563(01)01069-X

[JCS194571C77] Paoletti A. and Chang F. (2000). Analysis of mid1p, a protein required for placement of the cell division site, reveals a link between the nucleus and the cell surface in fission yeast. *Mol. Biol. Cell.* 11, 2757-2773. 10.1091/mbc.11.8.2757 10930468 PMC14954

[JCS194571C78] Polanshek M. M. (1977). Effects of heat shock and cycloheximide on growth and division of the fission yeast *Schizosaccharomyces pombe*. *J. Cell. Sci.* 23, 1-23. 893531 10.1242/jcs.23.1.1

[JCS194571C79] Rao K. V. and Cullen W. P. (1960). Streptonigrin, an antitumor substance. *Antibiot. Annu.* 1959-1960, 950-953. 14436228

[JCS194571C80] Rao K. V., Biemann K. and Woodward R. B. (1963). The structure of Streptonigrin. *J. Am. Chem. Soc.* 85, 2532-2533. 10.1021/ja00899a051

[JCS194571C81] Ribas J. C., Diaz M., Duran A. and Perez P. (1991). Isolation and characterization of *Schizosaccharomyces pombe* mutants defective in cell wall (1-3) beta-D-glucan*.* *J. Bacteriol.* 173, 3456-3462. 10.1128/jb.173.11.3456-3462.1991 1828464 PMC207959

[JCS194571C82] Rodriguez L., Laborda F. and Sentandreu R. (1979). Patterns of wall synthesis in *Saccharomyces cerevisiae*. *Curr. Microbiol.* 2, 293-297. 10.1007/BF02602862

[JCS194571C83] Russell P. and Nurse P. (1986). Cdc25+ functions as an inducer in the mitotic control of fission yeast. *Cell* 45, 145-153. 10.1016/0092-8674(86)90546-5 3955656

[JCS194571C84] Russell P. and Nurse P. (1987). Negative regulation of mitosis by wee1+, a gene encoding a protein kinase homolog. *Cell* 49, 559-567. 10.1016/0092-8674(87)90458-2 3032459

[JCS194571C85] Sahin N. (1995). Selective isolation and classification of novel thermotolerant Streptomycetes. *PhD thesis*, University of Newcastle, Newcastle upon Tyne, UK.

[JCS194571C86] Saka Y. and Yanagida M. (1993). Fission yeast cut5+, required for S phase onset and M phase restraint, is identical to the radiation-damage repair gene rad4+. *Cell* 74, 383-393. 10.1016/0092-8674(93)90428-S 8343962

[JCS194571C87] Sato M. and Toda T. (2010). Space shuttling in the cell: nucleocytoplasmic transport and microtubule organization during the cell cycle. *Nucleus* 1, 231-236. 10.4161/nucl.11443 21327068 PMC3027027

[JCS194571C88] Sembiring L. (2000). Selective isolation and characterization of Streptomycetes associated with the rhizosphere of the tropical legume *Paraserianthes falcataria* (L) Nielsen. *PhD thesis*, University of Newcastle, Newcastle upon Tyne, UK.

[JCS194571C89] Sentandreu R. and Lampen J. O. (1970). Biosynthesis of yeast mannan: inhibition of synthesis of mannose acceptor by cycloheximide. *FEBS Lett.* 11, 95-99. 10.1016/0014-5793(70)80500-2 11945458

[JCS194571C90] Shinmura K., Tarapore P., Tokuyama Y., George K. R. and Fukasawa K. (2005). Characterisation of centrosomal association-of nucleophosmin/B23 clinked to Crm1 activity. *FEBS Lett.* 579, 6621-6634. 10.1016/j.febslet.2005.10.057 16297385

[JCS194571C91] Simanis V. and Nurse P. (1986). The cell cycle control gene cdc2+ of fission yeast encodes a protein kinase potentially regulated by phosphorylation. *Cell* 45, 261-268. 10.1016/0092-8674(86)90390-9 3516412

[JCS194571C92] Snaith H. A. and Sawin K. E. (2003). Fission yeast mod5p regulates polarised growth through anchoring of tea1p at cell tips. *Nature* 423, 647-651. 10.1038/nature01672 12789340

[JCS194571C93] Stade K., Ford C. S., Guthrie C. and Weis K. (1997). Exportin 1 (Crm1p) is an essential nuclear export factor. *Cell* 90, 1041-1050. 10.1016/S0092-8674(00)80370-0 9323132

[JCS194571C94] Sugiura Y., Kuwahara J. and Suzuki T. (1984). DNA interaction and nucleotide sequence cleavage of copper-streptonigrin. *Biochem. Biophys. Acta.* 782, 254-261. 10.1016/0167-4781(84)90060-5 6329300

[JCS194571C95] Takeda T. and Chang F. (2005). Role of fission yeast myosin 1 in organisation of sterol-rich membrane domains. *Curr. Biol.* 15, 1331-1336. 10.1016/j.cub.2005.07.009 16051179

[JCS194571C96] Takeda T. Kawate T. and Chang F. (2004). Organization of a sterol-rich membrane domain by cdc15p during cytokinesis in fission yeast. *Nat. Cell. Biol.* 6, 1142-1144. 10.1038/ncb1189 15517003

[JCS194571C97] Takemoto A., Kawashima S. A., Li J. J., Jeffery L., Yamatsugu K., Elemento O. and Nurse P. (2016). Nuclear envelope expansion is crucial for proper chromosomal segregation during a closed mitosis. *J. Cell Sci.* 129, 1250-1259. 10.1242/jcs.181560 26869222 PMC4813296

[JCS194571C98] Tange Y., Hirata A. and Niwa O. (2002). An evolutionarily conserved fission yeast protein, Ned1, implicated in normal nuclear morphology and chromosome stability, interacts with Dis3, Pim1/RCC1 and an essential nucleoporin. *J. Cell Sci.* 115, 4375-4385. 10.1242/jcs.00135 12376568

[JCS194571C99] Thompson B. J. and Sahai E. (2015). MST kinases in development and disease. *J. Cell Biol.* 131, 1529-1538. 10.1083/jcb.201507005 PMC4576864 26370497

[JCS194571C100] Tsuchiya E., Yukawa M., Ueno M., Kimura K. and Takahashi H. (2010). A novel method of screening cell-cycle blockers as candidates for anti-tumor reagents using yeast as a screening tool. *Biosci. Biotechnol. Biochem.* 74, 411-414. 10.1271/bbb.90633 20139596

[JCS194571C101] Umenzawa H., Maeda K., Takeuchi T. and Okami Y. (1966). New antibiotics, bleomycin A and B. *J. Antibiot.* 19, 200-209. 5953301

[JCS194571C102] Verde F., Mata J. and Nurse P. (1995). Fission yeast cell morphogenesis: identification of new genes and analysis of their role during the cell cycle. *J. Cell Biol.* 131, 1529-1538. 10.1083/jcb.131.6.1529 8522609 PMC2120658

[JCS194571C103] Wachtler V., Rajagopalan S. and Balasubramanian M. K. (2003). Sterol-rich plasma membrane domains in the fission yeast *Schizosaccharomyces pombe*. *J. Cell. Sci.* 116, 867-874. 10.1242/jcs.00299 12571284

[JCS194571C104] Wang W., Budhu A., Forgues M. and Wang X. W. (2005). Temporal and spatial control of nucleophosmin by the Ran-Crm1 complex in centrosome duplication. *Nat. Cell. Biol.* 7, 823-830. 10.1038/ncb1282 16041368

[JCS194571C105] Whiffen A. J., Bohonos N. and Emerson R. L. (1946). The production of an antifungal antibiotic by *Streptomyces griseus*. *J. Bacteriol.* 52, 610-611. 16561221 10.1128/jb.52.5.610-611.1946 PMC518238

[JCS194571C106] Wood V., Gwilliam R., Rajandream M.-A., Lyne M., Lyne R., Stewart A., Sgouros J., Peat N., Hayles J., Baker S. et al., (2002). The genome sequence of *Schizosaccharomyces pombe*. *Nature* 415, 871-880. 10.1038/nature724 11859360

[JCS194571C107] Yamashita Y., Kawada S.-Z., Fujii N. and Nakano H. (1990). Induction of mammalian DNA topoisomerase II dependent DNA cleavage by antitumor antibiotic Streptonigrin. *Cancer Res.* 50, 5841-5844. 2168283

[JCS194571C108] Yanagida M. (1998). Fission yeast cut mutations revisited: control of anaphase. *Trends Cell Biol.* 8, 144-149. 10.1016/S0962-8924(98)01236-7 9695827

